# Brain Metabolic Changes with Longitudinal Transcutaneous Afferent Patterned Stimulation in Essential Tremor Subjects

**DOI:** 10.5334/tohm.565

**Published:** 2020-12-16

**Authors:** Abhijeet S. Barath, Aaron E. Rusheen, Hoon-Ki Min, Jeyeon Lee, Erika Ross, Sooyoon Shin, Adam Loudermilk, Bambi Wessel, Val J. Lowe, Kendal H. Lee, Charles D. Blaha

**Affiliations:** 1 Department of Neurologic Surgery, Mayo Clinic, Rochester, MN, US; 2 Mayo Clinic Graduate School of Biomedical Sciences, Mayo Clinic, Rochester, MN, US; 3 Medical Scientist Training Program, Mayo Clinic Alix School of Medicine, Mayo Clinic, Rochester, MN, US; 4 Department of Radiology, Mayo Clinic, Rochester, MN, US; 5 Cala Health, Inc., Burlingame, CA, US; 6 Department of Physiology and Biomedical Engineering, Mayo Clinic, Rochester, MN, US

**Keywords:** essential tremor, noninvasive stimulation, transcutaneous afferent patterned stimulation, positron emission tomography, deep brain stimulation

## Abstract

**Background:**

Non-invasive peripheral nerve stimulation, also referred to as transcutaneous afferent patterned stimulation (TAPS), reduces hand tremor in essential tremor (ET) subjects. However, the mechanism of action of TAPS is unknown. Here, we investigated changes in brain metabolism over three months of TAPS use in ET subjects.

**Methods:**

This was an interventional, open label, single group study enrolling 5 ET subjects. They received 40 minutes of TAPS treatment twice daily for 90 days. Brain metabolic activity and tremor severity were measured using 18F-fluorodeoxyglucose (FDG) PET/CT, and the Tremor Research Group Essential Tremor Rating Assessment Scale (TETRAS), respectively, at baseline and after 90 days. Tremor power and frequency was measured before and after all TAPS sessions using an onboard three-axis accelerometer.

**Results:**

FDG PET/CT revealed areas of hypermetabolism in ipsilateral cerebellar hemisphere and hypometabolism in contralateral cerebellar hemisphere following 90 days of TAPS treatment, compared to day one (uncorrected p value <0.05). Paired pre-post kinematic measurements over 90 days showed significantly decreased tremor power (p < 0.0001) but no change in tremor frequency. The TETRAS score on day 1 decreased from 6.5 ± 2.5 to 4.1 ± 1.8 following TAPS (p = 0.05). The pre-post TETRAS scores on day 90: 4.9 ± 1.5 and 4.1± 1 were lower than pre-TAPS TETRAS score on day 1 (p = 0.14 and 0.05, respectively).

**Conclusions:**

Our results suggest that longitudinal TAPS of the median and radial nerves modulates brain metabolism in areas instrumental to motor coordination and implicated in ET. Clinically, TAPS reduced tremor power, but had no effect on tremor frequency. This study paves the way for comprehensive studies in larger cohorts to further elucidate the mechanism of TAPS.

**Highlights:**

Non-invasive peripheral nerve stimulation, also referred to as transcutaneous afferent patterned stimulation (TAPS), reduces hand tremor in essential tremor subjects. Longitudinal TAPS therapy alters cerebellar metabolism, which can be a cause or consequence of tremor reduction. Cerebellar-premotor region connectivity may play a role in the anti-tremor effects of TAPS.

## Introduction

Essential tremor (ET) is the most common movement disorder, affecting an estimated 7 million Americans [1]. ET patients suffer from disabling shaking of their hands and, less commonly, head, voice, tongue, leg, and trunk which negatively impacts their quality of life [2,3,4]. Pharmacotherapy is the mainstay for treatment of ET. However, the first-line agents are ineffective in 30–70% patients due to lack of response, adverse effects, and development of drug tolerance [5,6,7,8]. These patients are candidates for invasive neurosurgical interventions, including deep brain stimulation (DBS) and high intensity focused ultrasound (HIFU) thalamotomy. While effective [9,10,11,12,13,14], these interventions are associated with the general risks of surgery, as well as stimulation-induced adverse effects (e.g., paresthesia, dystonia, dysarthria, gait disturbances) [14,15,16]. They are also expensive and not widely available.

More recently, non-invasive electrical stimulation of peripheral nerves at the wrist has been shown to reduce hand tremor in ET subjects [17,18]. These transcutaneous electrical pulses are programmed to alternate between radial and median nerves in a pattern resembling tremor frequency, earning it the name transcutaneous afferent patterned stimulation (TAPS). Improvement in tremor and quality of life has been demonstrated with both acute, as well as, repeated use of TAPS [17,18,19]. The recently published PROSPECT trial has shown the efficacy of longitudinal TAPS therapy, as well as a reduction in baseline pre-TAPS tremor scores over three months of home use [19]. However, the mechanism of action of TAPS and whether its long-term use modulates brain plasticity remains unknown. Therefore, in this study we sought to determine whether and in what ways daily TAPS therapy over a three month period may affect brain metabolism. We also examined changes in tremor power, frequency, and clinical tremor severity over this period. The preliminary results of our study are presented.

## Methods

### Standard Protocol Approval, Registration, and Patient Consent

This study was registered with ClinicalTrials.gov (NCT03778060). The clinical protocol and informed consent form were approved by the Mayo Clinic Institutional Review Board (IRB 18-006984). Written informed consent was obtained from all participants prior to enrollment in the study.

### Subjects

This was a pilot study with 5 subjects. Subjects were screened for study eligibility through face-to-face interviews. Subjects were eligible for this study if they were ≥21 years and approved for DBS surgery by the Mayo Clinic Deep Brain Stimulation Committee for treatment of ET. Subjects were excluded if they had moderate to severe ethanol dependence or had an implanted pacemaker, defibrillator, or deep brain stimulator. A detailed description of the inclusion and exclusion criteria is provided in the supplementary information.

### Study Protocol

After a minimum 4 hour overnight fast, on day 1 of the study, the subjects were given an 8 mCi 18F-fluorodeoxyglucose (FDG) intravenous injection. After 30 minutes of FDG uptake, a 1 min Computed Tomography (CT) scan of the head was acquired for attenuation correction and anatomical co-registration and thereafter a 15 min FDG acquisition Positron Emission Tomography (PET) scan of the subject’s brain was obtained on a GE DMI PET/CT scanner (GE Healthcare, Waukesha, WI). Immediately following the PET/CT session, The Essential Tremor Rating Assessment Scale (TETRAS), a subscale version that included upper limb tremor assessment and the Archimedes spiral drawing task was conducted (details in tremor severity assessment section) [17,20,21,22,23].

The subjects were then fitted with a wrist-worn TAPS device (Cala Health Inc., Burlingame, CA). The hand with more severe tremor (or the dominant hand if both hands had equal tremor severity), as determined by the TETRAS and Archimedes spiral task, was chosen for stimulation. An accelerometer on-board the device measured the subject’s tremor frequency and power while the subject performed a forward postural arm hold task. This frequency was then incorporated into the therapeutic stimulation waveform. The device had two working stimulation electrodes positioned over the median and radial nerves on the anterior surface of the wrist, and a single counter-electrode positioned on the posterior surface of the wrist. Stimulation consisted of a series of charge balanced biphasic pulses delivered at a frequency of 150 Hz, 300 µs pulse width, and 50 µs inter-pulse period [17]. The stimulation was alternated between the median and radial nerve at a frequency equal to tremor frequency (i.e., for a measured 5 Hz tremor frequency, stimulation was applied over the median nerve for 100 msec, then alternated to be applied over the radial nerve for 100 msec). Stimulation amplitude was determined by increasing the stimulation level by 0.25 mA steps until the subject reported a sensation of the stimulus in the hand. Final stimulation amplitude was chosen to be the highest level of stimulation that the subject found comfortable. Subjects received stimulation at that level during a 40-min stimulation session and for every session thereafter. Immediately following the initial 40-minute session, TETRAS and Archimedes spiral drawing tasks were repeated.

Subjects then received device training and instructions by on-site study personnel for conducting daily home stimulation sessions, and were instructed to perform the home stimulation sessions twice daily for three months. Adverse events were monitored and reported to the Mayo Clinic IRB committee. At the end of the three-month study period, the subjects returned to the office. Their medication and vitals were reviewed. The subjects then underwent a FDG PET/CT session identical to that conducted on day 1 of the study. Thereafter, TETRAS and Archimedes spiral drawing tasks were conducted before and after 40 minutes of stimulation with the device.

### Tremor Severity Assessment

Tremor severity in the upper limbs was assessed using tremor power analysis, TETRAS subscores and Archimedes spiral drawing tasks. The kinematic data was obtained using a three-axis accelerometer on board the device when the subjects performed a forward postural hold task before and after TAPS. The sum of power spectral density (PSD) of tremor frequency band was computed from the accelerometer data before and after each TAPS session to compute tremor power. See supplementary information for details of how PSD was calculated and filtered.

TETRAS ratings were assessed independently by two observers on a scale of 0–4 for each task [23]. The ratings for the forward horizontal reach posture and lateral “wing beating” posture (each held for 20 sec), and finger-nose-finger testing (executed three times) was summed to obtain the final scores. Tremor severity was also assessed using the Archimedes spirals drawing task. For this, subjects drew an Archimedes spiral that approximately filled ¼ of an unlined page of standard (letter) paper (approximate size 10 × 10 cm) after demonstration by the examiner. See supplementary information (Tables S1 and S2) for details of TETRAS and Archimedes spiral score assignment.

### Data and Statistical Analysis

To identify changes in patterns of metabolism between baseline, and subsequent scans we utilized Statistical Parametric Mapping-12 (SPM12) running on MATLAB R2016b (Mathworks, Natick, MA) and AFNI software package (https://afni.nimh.nih.gov/afni). All the FDG images were co-registered to a population-optimized FDG PET templates from the Mayo Clinic Adult Lifespan Template (MCALT) [24] using series of affine and nonlinear registration steps. The images were normalized to the pons, followed by spatial smoothing with isotropic kernel (full width at half maximum = 6mm). Group statistical comparisons of day 90 scan was compared to the day 1 scan using a paired t-test in a voxel-by-voxel manner [25]. The age and gender was used as a covariate. Voxels with a p value < 0.05 (uncorrected) and cluster size > 40 voxels were used to determine significant differences. For the interpretation of cerebellar metabolism change, a cerebellar parcellation map was adopted.(26) To apply this atlas to our data, the atlas was projected onto the MCALT space.

Clinical efficacy was measured as the change in the tremor power (summed PSD), TETRAS, and Archimedes spiral drawing scores following TAPS compared with pre-TAPS. The Wilcoxon matched pair rank sum test was used to compare the following parameters for individual subjects: (1) summed PSD before and after TAPS over the 90 days of use, (2) tremor frequency before and after TAPS for the first 14 sessions, and (3) the pre-TAPS tremor frequency for the first and last 14 sessions. A paired t-test was used to investigate the following differences in TETRAS and Archimedes spiral ratings for all 5 subjects: (1) pre- and post-TAPS on day 1 and 90, to assess acute therapeutic effects and (2) pre-TAPS on day 1 compared to pre- and post-TAPS on day 90, to assess cumulative therapeutic effects with long-term use.

We also evaluated the PET-clinical tremor relationship by looking at Pearson correlation coefficient for clinical tremor reduction (Δ pre-TAPS TETRAS) and change of FDG standardized uptake value ratio (Δ SUVR) for the cerebellar sites. Study compliance for individual subjects was determined based on device data indicating completed sessions throughout the three month at-home period. Compliance was calculated based on twice a day usage during enrollment period (i.e., percent compliance = 100 * (number of total completed sessions/(2 * number of enrollment days)).

## Results

Five subjects (3 males, 2 females) with medically intractable ET, approved to undergo DBS surgery by the Mayo Clinic DBS Committee, participated in the study. The mean age of the study participants was 70.2 ± 5.2 years (mean ± 1 SD). The duration of their disease ranged from 10 to 57 years (mean 32 ± 16.8 years). The participants were enrolled for 89.2 ± 4.8 days and completed 136.2 ± 41.3 TAPS sessions. Their compliance was found to be 77.3 ± 25.6 % (Table 1).

**Table 1:** **Subject demographics and compliance summary.** Abbreviations: Avg, average; SD, standard deviation; BL, bilateral; L, left; R, right.

	Age (yrs)	Disease duration (yrs)	Tremor frequency (Hz)	Medications	Stimulated hand	Enrollment days	Completed TAPS sessions	Compliance^#^ (%)

1	75	25	BL: 5.3–5.4	Propranolol and primidone	Right	86	156	90.70
2	62	30–40	BL: 4.5–5.0	Propranolol	Right	88	131	74.43
3	74	57	L: 3.8–4.2 R: 4.0	Propranolol, primidone, gabapentin, and acetazolamide	Left	97	67	34.54
4	71	30–35	BL: 5.2–5.8	Primidone and gabapentin	Right	85	171	100.00
5	69	10–12	BL: 4.9–5.3	None	Right	90	156	86.67
**Avg**	70.2					89.2	136.2	77.3
**SD**	5.2					4.8	41.3	25.6

^#^ % Compliance is based on twice a day use during enrollment days = (100 * No. of completed TAPS sessions)/(2 * No. of enrollment days).

**Figure 1:**
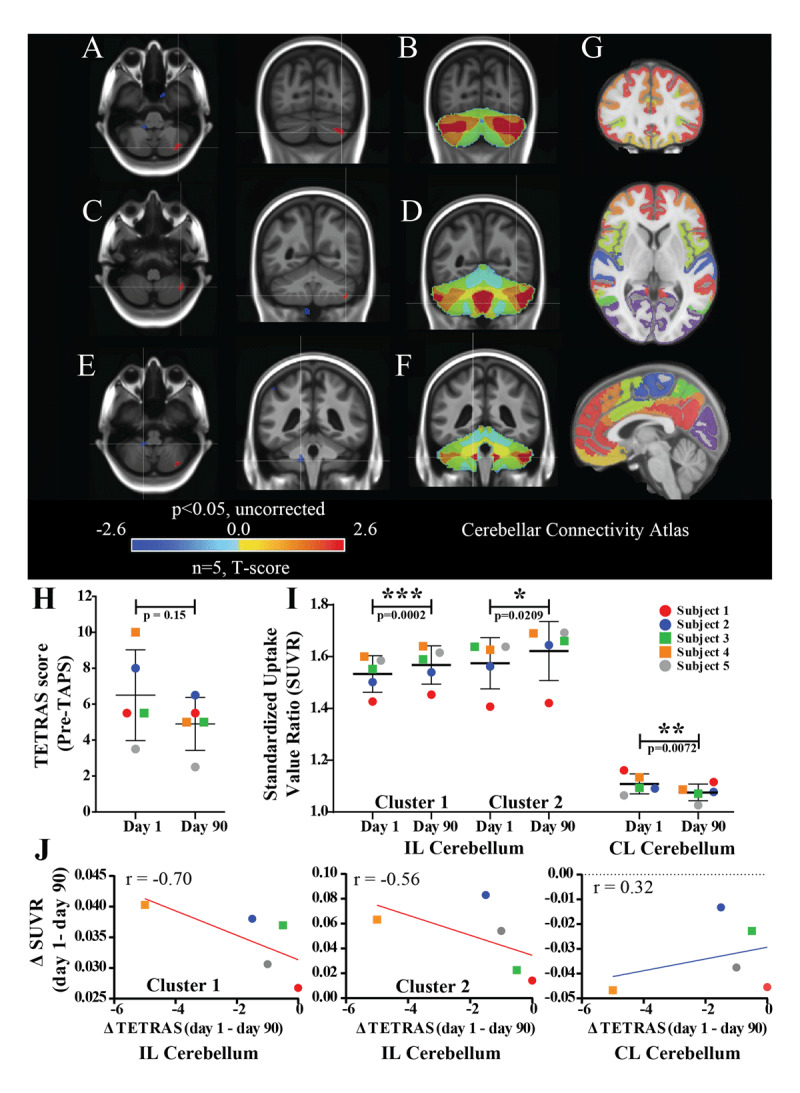
**PET results and PET-tremor correlation: (A–G)** PET imaging of TAPS-induced brain regional activity at day 90, compared to day 1, averaged across 5 subjects with essential tremor (p<0.05, uncorrected), showing **(A, C)** hypermetabolic clusters in ipsilateral cerebellar hemisphere and **(E)** a hypometabolic cluster in contralateral cerebellar hemisphere. **(B, D, F, G)** Cerebellar connectivity atlas showing cerebral-cortical connections for different cerebellar regions- the red color for example shows connection to frontal pole and medial frontal cortex and the orange color shows connection to frontal and parietal cortex, including premotor region. **(H)** Pre-TAPS clinical tremor scores (TETRAS) on day 1 and day 90 for all 5 ET patients. **(I)** Absolute change of SUVR for the three cerebellar clusters shown in (A),(C) and (E) at day 90 compared to day 1. **(J)** Correlation between change of tremor scores and SUVR for the cerebellar clusters from day 1 to 90. Abbreviations: CL, contralateral; ET, essential tremor; IL, ipsilateral; PET, positron emission tomography; TAPS, transcutaneous afferent patterned stimulation; TETRAS, Tremor Research Group Essential Tremor Rating Assessment Scale; SUVR, standardized uptake value ratio. p-values: * <0.05, ** <0.01, *** <0.0005.

PET imaging revealed two clusters of increased glucose metabolism in the ipsilateral cerebellar hemisphere and one cluster of reduced glucose metabolism in the contralateral cerebellar hemisphere (p < 0.05, uncorrected) at day 90 compared to day 1 (Figure 1A, C and E, Table 2). Other ipsilateral regions, including pre- and post-central regions and middle occipital lobe and contralateral regions, including insula, cuneus, anterior cingulate, and inferior parietal cortex, exhibited clusters of decreased glucose metabolism (p < 0.05, uncorrected, Table 2). The changes in SUVR for both ipsilateral hypermetabolic clusters (p = 0.0002 and 0.0209, respectively) and the contralateral hypometabolic cluster (p = 0.0072) were statistically significant (Figure 1I). The functional cerebellar atlas showed connectivity of ipsilateral hypermetabolic cerebellar clusters to contralateral prefrontal and parietal regions, including the premotor area. The contralateral hypometabolic cerebellar cluster also showed connectivity with the frontal pole, medial frontal cortex, and parts of the medial parietal cortex. (Figure 1B, D, F and G).

**Table 2:** **PET results:** Regions showing statistically significant changes in 18F-fluorodeoxyglucose (FDG) uptake at day 90 compared to day 1 following longitudinal TAPS therapy (p<0.05, uncorrected). Abbreviations: CL, contralateral; IL, ipsilateral.

Region	Maximum T-score	Cluster size	Talairach coordinates		

			*x*	*y*	*z*

IL Cerebellum crus II cluster 1	3.45	145	34.5	–70.5	–39
IL Cerebellum crus II cluster 2	3.29	61	39	–54	–45
IL Precentral area	–2.65	40	15	–21	52.5
IL Postcentral area	–3.05	85	60	–13.5	10.5
IL Middle occipital	–3.29	54	24	–81	1.5
CL Cerebellum Lobule IX	–2.73	40	–13.5	–42	–33
CL Insula	–2.77	98	–34.5	–12	10.5
CL Cuneus	–3.1	47	–7.5	–79.5	10.5
CL Anterior cingulate	–3.26	171	–1.5	45	0
CL Inferior parietal	–3.77	40	–43.5	–46.5	43.5

**Figure 2:**
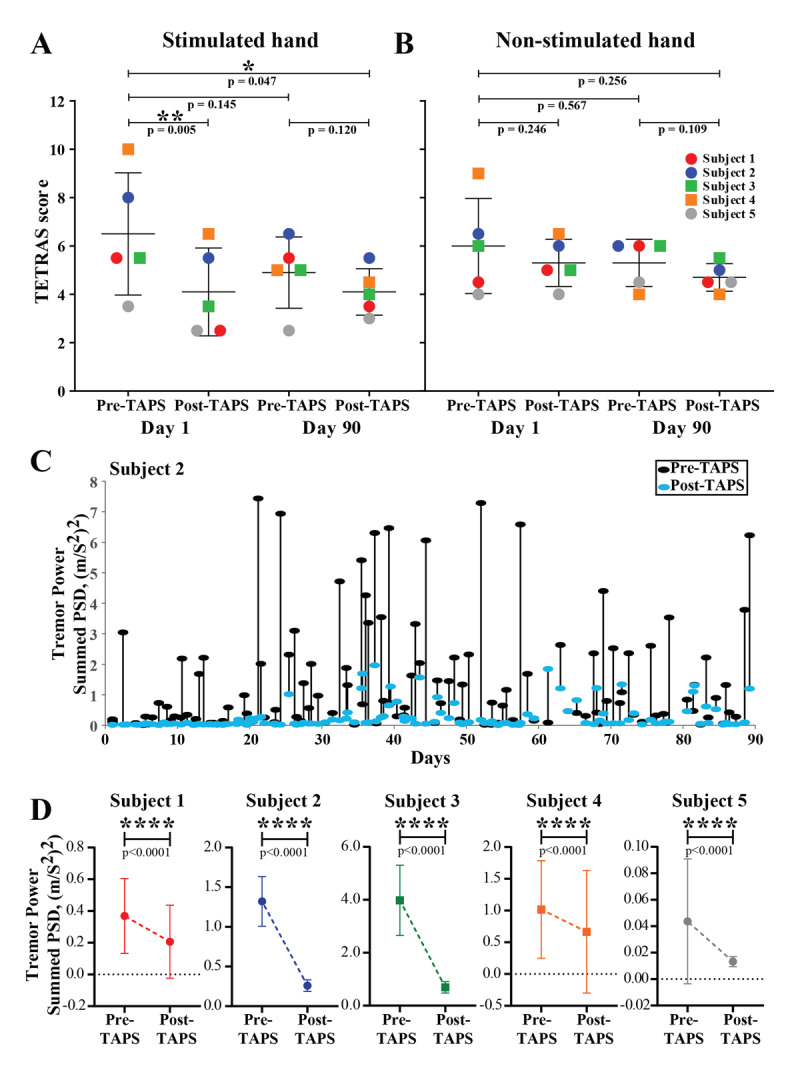
**Tremor assessment:** Clinician-rated change in TETRAS scores for both **(A)** stimulated and **(B)** non-stimulated hand at day 1 and day 90 (n = 5). **(C)** Tremor power before (black dot) and after (blue dot) each stimulation session (connected with a line) followed over a 90 days period for one of our representative subjects showing a decrease in tremor power after most stimulation sessions. Other subjects’ data is provided in the supplementary information (Figure S1). **(D)** Change in pre- and post-TAPS tremor power (pair-wise comparison) for individual subjects over the 90 days of use. The difference in Y-scales indicates inter-subject variability in tremor severity. Abbreviation: TAPS, transcutaneous afferent patterned stimulation; TETRAS, Tremor Research Group Essential Tremor Rating Assessment Scale. p-values: * <0.05, ** <0.01, **** <0.0001.

On day 1 (first visit), the mean TETRAS scores for the stimulated hand, before and after TAPS, were found to be 6.5 ± 2.5 and 4.1 ± 1.8, respectively (mean ± SD; p = 0.005, Figure 2A). The Archimedes spiral scores pre- and post-TAPS were 2.6 ± 0.5 and 2.0 ± 0.0, respectively (p = 0.07). Similarly, on day 90 (final visit), the mean TETRAS scores for the stimulated hand were found to be 4.9 ± 1.5 and 4.1 ± 1.0 (p = 0.12) before and after TAPS (Figure 2A). The Archimedes spiral scores changed from 2.8 ± 0.4 to 2.0 ± 1.0 (p = 0.09). The TETRAS scores for the non-stimulated hand are summarized in Figure 2B.

The paired pre-post tremor power analysis for individual subjects showed a consistent decrease (p < 0.0001) following TAPS use across the entire duration of use (Figure 2C and D). An overall median tremor power reduction of 72.6 ± 14.5% was noted (supplementary table S5). Figure 2C shows the tremor power recordings for a representative subject (subject 2) over the 3 month treatment period (see supplementary information Figure S1 for tremor power recordings of other subjects). No statistically significant changes were observed in tremor frequency with acute or long-term TAPS use (p =1.00 and 0.52, respectively; see supplementary information Table S3).

The Pearson correlation coefficient ‘r’ for ΔSUVR (day 1–day 90 for cerebellar clusters) and Δ pre-TAPS TETRAS scores (day 1–day 90) was found to be –0.70 and –0.56 for the hypermetabolic clusters in the ipsilateral cerebellar hemisphere and 0.32 for the hypometabolic cluster in the contralateral cerebellar hemisphere (Figure 1J).

## Discussion

### Longitudinal TAPS therapy modulates cerebellar metabolism

Our preliminary results demonstrate clusters of increased metabolism in the cerebellar hemisphere ipsilateral to the side of stimulation and a hypometabolic region in the contralateral cerebellar hemisphere following 90 days of TAPS therapy (p < 0.05, uncorrected, Figure 1A, C and E). During this period we also observed a median tremor power reduction of 72.6 ± 14.5% (n = 5) across subjects. The metabolic changes observed in the cerebellum could be due to the direct effect of TAPS leading to tremor reduction, a consequence of tremor reduction, or an effect of TAPS unrelated to tremor or noise. The third possibility is least likely as- 1) cerebellum is a well-established and important node in the tremor circuitry [27,28,29,30,31], 2) we observed the cerebellar changes most consistently in our data set regardless of the reference brain region or method chosen for PET analysis (further discussed below), and 3) a moderate to strong correlation was found between Δ pre-TAPS TETRAS scores and ΔSUVR for the two hypermetabolic clusters in ipsilateral cerebellar hemisphere (Figure 1J). However, with our current study design it is not possible to tease out whether the tremor reduction is a cause or a consequence of cerebellar metabolic change.

In either case, our findings are in line with cerebellar activation observed ipsilateral to the side of tremor control with ventral intermediate (ViM) nucleus DBS for ET [32]. This cerebellar activation was also shown to have the strongest association with long-term therapeutic effects of VIM DBS [32]. Similarly in our data set, we observed a moderate to strong correlation (Pearson’s r –0.7 and –0.56) between change of SUVR in cerebellar hypermetabolic clusters and change in clinical tremor scores recorded just before PET (pre-TAPS TETRAS) on the respective days (Figure 1J). However, our observations are different from the bilateral cerebellar inactivation (reduction in regional cerebral blood flow, rCBF) noted with ethanol intake in alcohol responsive ET patients [33]. Ethanol likely acts by enhancing inhibitory GABAergic (gamma-aminobutyric acid) transmission [34] in brains of ET patients who are known to have reduced GABA receptor concentrations in their cerebellar dentate nuclei [35]. It is possible that increased glucose utilization in ipsilateral cerebellar cortex with TAPS therapy could indicate increased Purkinje cell activity. Purkinje cells send GABAergic efferents to dentate nuclei and their loss has been observed in ET patients [36]. Further studies showing direct activation of Purkinje cells and/or increase GABA release with TAPS therapy are required to confirm this hypothesis.

We also observed decreased metabolism in other brain regions, including ipsilateral pre- and post-central areas, middle occipital lobe, and contralateral regions, including insula, cuneus, anterior cingulate, and inferior parietal cortex (p < 0.05, uncorrected). The functional significance of these observations with respect to tremor is unclear. We continue our investigations in a larger cohort of patients.

### Cerebellar-premotor region connectivity may play a role in mechanism of TAPS

The cerebellar connectivity atlas [26] revealed connections of ipsilaterally activated cerebellar regions with contralateral prefrontal and parietal regions, including the premotor area (Figure 1B, D, F and G). Magnetoencephalography studies have revealed that ET patients exhibit contralateral premotor activity that correlates with tremor frequency [37]. Similarly, involvement of the contralateral primary motor cortex, thalamus, brainstem, and ipsilateral cerebellum has also been observed [27,37,38]. Current theory suggests the presence of multiple tremor pattern generators throughout the aforementioned structures which entrain with one another to produce the symptoms of tremor [39,40]. Therefore, potential Purkinje cell activation in the cerebellar cortex by TAPS could reduce this entrainment by increasing inhibitory GABAergic drive. However, we did not observe a statistically significant signal in contralateral premotor or motor cortices and the other nodes of the tremor network. This could be due to low sensitivity resulting from the small sample size and preliminary nature of our work. The cortical and cerebellar activating effects seen with VIM DBS are possibly related to orthodromic and antidromic activation, respectively, of thalamo-cortical and cerebello-thalamic projections [41,42]. Whether the mechanism of TAPS also involves activation of cerebello-thalamo-cortical pathways remains to be determined.

### TAPS consistently reduced tremor

In addition to findings from PET, we found a consistent and statistically significant (p < 0.0001) reduction in paired pre-post-tremor power for all subjects across the 90 days of TAPS use regardless of their compliance and initial tremor scores (Figure 2C and D, supplementary figure S1). The median tremor power reduction was found to be 72.6%. This is in line with the findings from a large multi-center trial (PROSPECT) of TAPS therapy [43]. We also noted an acute, statistically significant decrease in TETRAS scores on day 1, but not on day 90, following a 40 min TAPS session (Figure 2A). A trend was noted for decreased pre-TAPS tremor score at day 90 compared to day 1. However, unlike the PROSPECT study [43] it did not reach statistical significance. These results must be seen in the light of considerable day to day variability seen in tremor as evident from the literature [44] and from our own longitudinal tremor power recordings (Figure 2C, supplementary figure S1). Also, this study was not powered to detect differences in clinical tremor outcomes (TETRAS scores) which has already been demonstrated by others using variants of this device in both sham-control and longitudinal home use settings [17,18,43].

### Limitations

This study had a number of limitations. First, this was an open-label study with a relatively small sample size and without a sham control. To assess the extent to which placebo effects may contribute to the outcomes, a sham control would be preferable. However, successfully facilitating a comparable blind mimicking the device’s therapeutic sensation with at-home long-term use is challenging. Small sample size does not allow the results to survive multiple correction or region of interest (ROI) analysis. Second, both the PET/CT and the clinical tremor ratings reflect a snapshot of time in the disease process. Fluctuations in ET severity on a day to day basis are well known and may be a potential source of bias in our results [44]. Third, we choose the pons as reference as it was expected to be less affected by TAPS compared to cerebellum. However, the pons is also within the cortical-thalamic-cerebellum tremor circuit, therefore future work is needed to define a better reference region. As such, we continue to evaluate the mechanism of TAPS in a larger cohort to confirm our preliminary findings.

## Conclusion

TAPS of radial and median nerves may improve tremor via modulation of neuronal/glia activity in the cerebellum and other brain regions associated with the tremor circuit in subjects with ET. This preliminary study identifies several anatomical areas within the brain which may be related to the therapeutic effect of this therapy or could be chance associations. Continuation of this study and future invasive studies in patients and animal models of ET would shed further light on the mechanism of TAPS.

## Additional File

The additional file for this article can be found as follows:

10.5334/tohm.565.s1 Supplementary Information. Detailed inclusion/exclusion criterion and tremor data.
