# Supplementary material for: Brain Metabolic Changes with Longitudinal Transcutaneous Afferent Patterned Stimulation in Essential Tremor Subjects
Source: Tremor Other Hyperkinet Mov (N Y). 2020 Dec 16;10:52. doi: 10.5334/tohm.565 (PMC7747758; doi:10.5334/tohm.565)
Supplement: Supplementary Information. — Detailed inclusion/exclusion criterion and tremor data. [file tohm-10-1-565-s1.pdf]

Title: Brain Metabolic Changes with Longitudinal Transcutaneous Afferent Patterned Stimulation in Essential Tremor Subjects

Supplementary Information

Inclusion and exclusion criterion-

Key inclusion criterion were 1) age ≥21 years; 2) approved for DBS surgery at Mayo Clinic for deep brain stimulation treatment of essential tremor; 3) competent and willing to provide signed, informed consent to participate in the study; 4) stable dose of tremor medications, if applicable, for 30 days prior to study entry; 5) stable dose of antidepressant medications, if applicable, for 90 days prior to study entry; 6) willing to comply with study protocol requirements including: remaining on a stable dosage of tremor and antidepressant medications, if applicable, during the duration of the study; 7) no significant alcohol or caffeine consumption or usage of Cala Two device within 8 hours of study visits; 8) women of child-bearing potential must be willing to have a pregnancy test within 48 hours of the optional PET/CT exams. For enrollment in the optional PET/CT study the subject must be enrolled in the Cala TWO study and meet all of the inclusion criteria and none of the exclusion criteria of the study.

Key exclusion criteria were 1) moderate to severe ethanol dependence as defined by the criteria outlined in the DSM-5 (score of 4 or higher); 2) implanted electrical medical device, such as a pacemaker, defibrillator, or deep brain stimulator; 3) previous thalamotomy procedure, including stereotactic thalamotomy, gamma knife radiosurgical thalamotomy, and focused ultrasound for the treatment of tremor; 4) suspected or

diagnosed epilepsy or other seizure disorder; 5) swollen, infected, inflamed areas, or skin eruptions, open wounds, or cancerous lesions of skin at stimulation site (wrist); 6) peripheral neuropathy affecting the tested upper extremity; 7) presence of any other neurodegenerative disease like Parkinson plus syndromes suspected on neurological examination. These include: multisystem atrophy, progressive supranuclear palsy, dementia with Lewy bodies, and Alzheimer’s disease; 8) anyone suspected to have the diagnosis of idiopathic Parkinson’s disease (PD). This includes excluding anyone with the presence of parkinsonian features including bradykinesia rigidity, or postural instability. Subjects who exhibit only mild resting tremor but no other symptoms or signs of PD may be included; 9) botulinum toxin injection for hand tremor within 6 months prior to study enrollment; 10) are participating or have participated in another interventional clinical trial in the last 30 days which may confound the results of this study, unless approved by the Principal Investigator; 11) significant alcohol or caffeine consumption within 8 hours of study enrollment, which may confound the results of the study, where significant caffeine is considered more than 95 mg (equivalent to a cup of coffee), and significant alcohol is considered more than 14 g (equivalent to 5 oz of wine, 12 oz of beer, or 1.5 oz of distilled spirits).; 12) subjects unable to communicate with the investigator and staff; 13) any health condition that in the investigator's opinion should preclude participation in this study; 14) pregnancy or anticipated pregnancy during the course of the study; 15) subjects will be excluded from the optional PET/CT study if they have one or more of the following conditions: subjects unable to lie down without moving for 15 minutes; subjects whose glucose tests >=200 mg/dL prior to the PET/CT exams; women of child-bearing potential showing a negative pregnancy test within 48

hours of the PET exams; claustrophobic patients unable to tolerate the PET/CT scans (no sedation can be offered).

Power Spectral Density and Data Filtering

Raw accelerometer data from each of the three axes were broken into five non-overlapping frames approximately 2.4 seconds in length. Spectra were calculated from each axis-frame and combined. Amplitude spectra were calculated using the discrete-time Fourier transform or PSD estimates were calculated using the periodogram with a Hann window. Then, spectra from each of the three axes for a given frame were combined using the L1 norm across each frequency bin:

$$L1 : P_{c,f} = \left|P_{x,f} + P_{y,f} + P_{z,f}\right|$$

where $P_{x,f}$ is the amplitude or power in frequency bin *f* in channel *x*.

Next, the combined spectrum for each of the four frames was examined for non-tremulous movement artifact. This was done by comparing the summed power in a low-frequency band (typically between 0 Hz and 2.73 Hz, but user-specified) and tremor band (typically 2.34 Hz surrounding the peak tremor frequency, which was constrained between 4 and 12 Hz, but tremor bandwidth and peak frequency search band were user-specified). If the frame's summed power in the tremor band was greater than the summed power in the low-frequency band, or both the summed tremor band and the summed low-frequency band were less than a heuristically determined threshold, the frame was considered free from movement. Movement-free frames were then averaged to yield a single spectrum.

To examine therapeutic effects, a kinematic metric was extracted from the movement-free combined spectra from each pre- and post-stimulation recording. The metric included amplitude/PSD at peak tremor frequency (± 1.5 Hz) between 4-12 Hz.

For each session, certain exclusion criteria were examined to determine if the metric from that session would be used in subsequent analyses. These criteria included at least one frame in pre- and post-stimulation recordings that was free from movement artifact (as defined by a threshold of 0.8 mm/s^2), a maximum of 60 minutes time between the end of stimulation and the post-stimulation recording, and a minimum of 120 minutes time in between a pre-stimulation recording and the conclusion of the previous stimulation session.

Tables

Tremor amplitude	TETRAS subscale score
No tremor	0
Barely visible	1
Visible but <1 cm	1.5
1-3 cm	2
3-5 cm	2.5
5-10 cm	3
10-20 cm	3.5
>20 cm	4

Table S1. Tremor amplitude and corresponding score for postural holds and kinetic tasks for the TETRAS subset used in this study.

Tremor amplitude	Archimedes spiral score
Normal spiral	0
Barely visible tremor	1
Obvious tremor	2
Portions of figure not recognizable	3
Figure not recognizable	4

Table S2. Ratings of Archimedes spiral scores used in this study.

Subject	Tremor frequency			
	First week		Last week	
	Pre-TAPS*	Post-TAPS*	Pre-TAPS*	Post-TAPS*
1	4.92	4.64	4.83	5.31
2	5.62	6.09	5.11	5.23
3	4.49	4.48	4.85	5.28
4	5.66	5.15	5.51	5.24
5	4.86	5.34	4.85	5.04
p-value	1.00		0.20	
	0.52			
	0.69			
**Average**	5.11	5.14	5.03	5.22
**SD**	0.51	0.64	0.29	0.11

*averaged over 14 sessions

Table S3. Effect of TAPS on tremor frequency.

Subject	Pre-TAPS tremor power [summed PSD in $(m/s^2)^2$]		Tremor power reduction#
	First Week*	Last week*	
1	0.15	0.07	53.33
2	0.12	0.65	-441.67
3	2.32	2.00	13.79
4	0.67	0.11	83.58
5	0.03	0.02	33.33
p-value	0.54		

*averaged over 14 sessions

Tremor power reduction = (pre-TAPS tremor power for first week - pre-TAPS power for last week)*100/ Pre-TAPS power for first week

Table S4. Effect of TAPS on pre-stimulation tremor power. Abbreviations: PSD, Power Spectral Density.

Subject	No. of sessions with TPR*	Median tremor power [summed PSD $(m/s^2)^2$] Pre-TAPS	Post-TAPS	*p*-value#	Median tremor power reduction** (%)
1	122	0.06	0.02	1.55E-08	66.7
2	125	0.56	0.08	9.19E-13	85.7
3	53	1.74	0.32	5.28E-08	81.6
4	114	0.19	0.04	9.03E-18	78.9
5	149	0.02	0.01	4.49E-09	50.0

* Following tremor power session data was excluded from analysis of tremor power:

- tremor power recording (TPR) with pre-measurement done less than 2 hours after previous TAPS offset
- tremor power recording done more than 1 hour after the current TAPS offset
- tremor power recording dominated with non-tremor, low frequency movement power in PSD

\# Wilcoxon matched pair rank sum test between all paired pre-post sessions for individual subjects.

** Median tremor power reduction = median of '(pre-TAPS tremor power - post-TAPS power)*100/pre-TAPS power' across all sessions per subject.

Table S5. Tremor power summary for all five subjects. The tremor power data (tremor power) was obtained using a three-axis accelerometer onboard the device. Abbreviations: PSD, power spectral density.

Figures

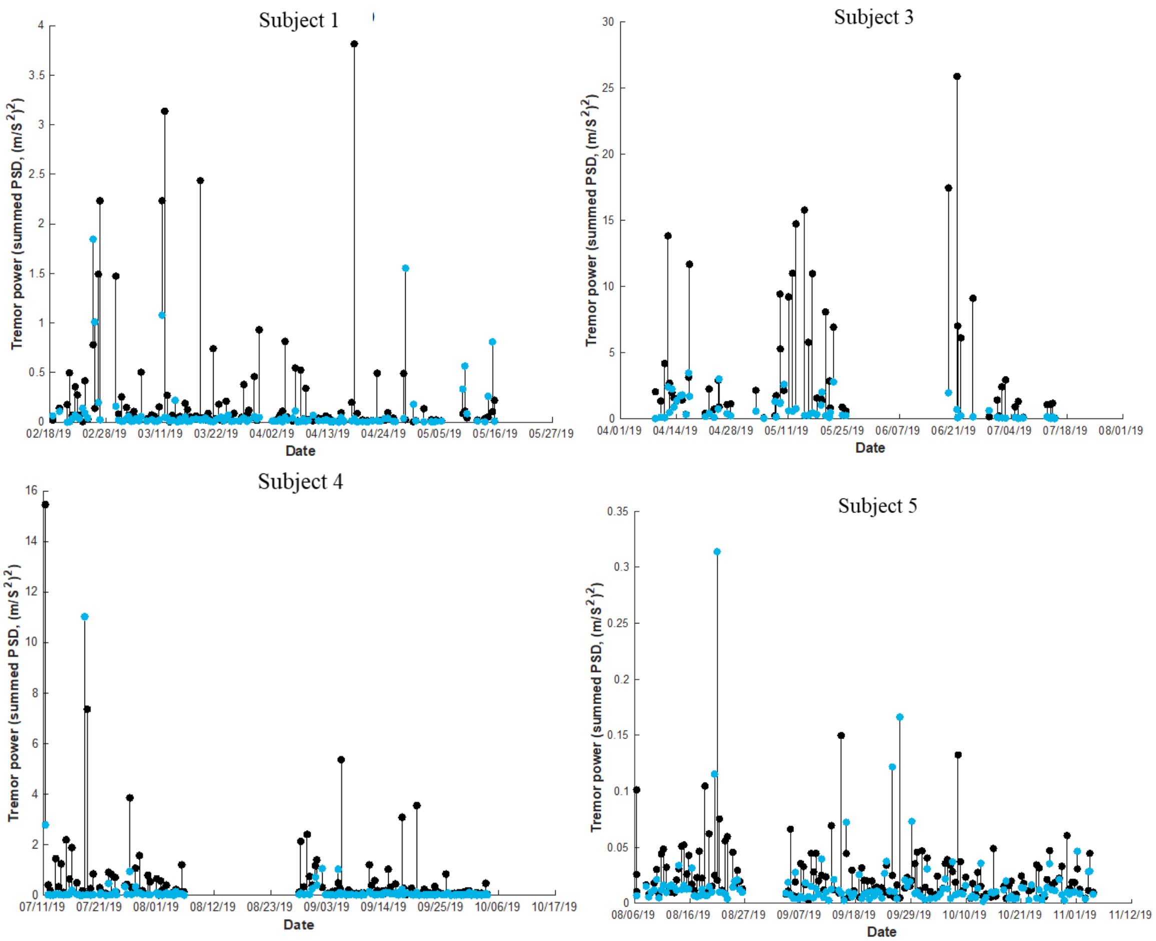

Figure S1. Paired pre- and post-TAPS tremor power for all subjects over the study period. Black dots show pre-TAPS tremor power, blue dots show post-TAPS tremor power.
